# Identification and distribution of microsymbionts associated with soybean nodulation in Mozambican soils^☆^

**DOI:** 10.1016/j.syapm.2018.05.003

**Published:** 2018-09

**Authors:** Cynthia Gyogluu, Sanjay K. Jaiswal, Stephen Kyei-Boahen, Felix D. Dakora

**Affiliations:** a Department of Crop Sciences, TshwaneUniversity of Technology, Pretoria, South Africa; b Department of Chemistry, Tshwane University of Technology, Pretoria, South Africa; c International Institute of Tropical Agriculture, Nampula, Mozambique

**Keywords:** Phylogeny, Horizontal gene transfer, Biogeography, Biological nitrogen fixation, Mozambique, CCA

## Abstract

Indigenous soybean rhizobial strains were isolated from root nodules sampled from farmers’ fields in Mozambique to determine their identity, distribution and symbiotic relationships. Plant infection assays revealed variable nodulation and symbiotic effectiveness among the 43 bacterial isolates tested. Strains from Ruace generally promoted greater whole-plant growth than the others. 16S rRNA-RFLP analysis of genomic DNA extracted from the rhizobial isolates produced different banding patterns, a clear indication of high bacterial diversity. However, the multilocus sequence analysis (MLSA) data showed alignment of the isolates with *B. elkanii* species. The 16S rRNA sequences of representative soybean isolates selected from each 16S rRNA-RFLP cluster showed their relatedness to *B. elkanii,* as well as to other *Bradyrhizobium* species. But a concatenated phylogeny of two housekeeping genes (*glnII* and *gyrB)* identified the soybean nodulating isolates as *Bradyrhizobium,* with very close relatedness to *B. elkanii.* The *nifH* and *nodC* sequences also showed that the majority of the test soybean isolates were closely related to *B. elkanii*, albeit the inconsistency with some isolates. Taken together, these findings suggest that the *B. elkanii* group are the preferred dominant microsymbiont of soybean grown in Mozambican soils. Furthermore, the distribution of soybean rhizobia in the agricultural soils of Mozambique was found to be markedly influenced by soil pH, followed by the concentrations of plant-available P and Mn. This study suggested that the identified isolates TUTMJM5, TUTMIITA5A and TUTLBC2B can be used as inoculants for increased soybean production in Mozambique.

## Introduction

Interest in soybean cultivation in Africa is increasing due to its high protein content of grain for human consumption and for use as animal feed. In Mozambique, there is an increasing demand for soybean, driven largely by the poultry industry and for exportation. This has led to many farmers switching to soybean production [42], as it offers them an income opportunity for their livelihood [19]. Soybean cultivation in Mozambique is dominated by smallholder farmers who use little or no inputs. The inaccessibility of rhizobial inoculants is a major factor contributing to low soybean yields, which were about 450 kg/ha in 2004, and 1100 kg/ha in 2012 [2], [12]. The increase was due to the application of inputs such as bacterial inoculants and P-fertilisers. Inoculating soybean with a suitable rhizobial strain can increase plant growth, symbiotic performance and grain yield [7], [14]. The breeding of TGx soybean genotypes was one way to reduce inoculant use by smallholder farmers [24]. Tropical Glycine cross (TGx) consists of soybean cultivars developed by breeders at the International Institute for Tropical Agriculture (IITA), Nigeria, that are freely nodulated by indigenous rhizobia in African soils. However, inconsistencies in nodulation and N_2_ fixation have been reported for the performance of these genotypes across the continent [30], [31], [33], [34], [35]. These inconsistencies suggest that in soils where effective indigenous rhizobia are lacking, inoculation may still be needed to improve grain yield. For the sustainable soybean production in Mozambique, a better understanding is needed of the diversity/phylogeny, distribution and effectiveness of the native soybean microsymbionts [28]. Knowledge of the rhizobial ecology is also needed in order to develop long-term strategies for the inoculation of soybean in Mozambique. Elsewhere in the world, diverse fast- and slow-growing bacteria belonging to the genera *Bradyrhizobium*, *Sinorhizobium* (*Ensifer*) and *Mesorhizobium* have been reported to effectively establish N_2_-fixing symbiosis with soybean [22], [46], [48]. Recently, Gyogluu et al. [14], [15] found a marked effect of *Bradyrhizobium* inoculation on plant growth, N content, %Ndfa (nitrogen derived from the atmosphere), amount of N fixed and grain yield when compared to uninoculated plants. However, little is known about the diversity, distribution and symbiotic effectiveness of native soybean rhizobia of Mozambique. Also, little is known of the effect of environmental factors on the distribution of these indigenous soybean rhizobia. In this study, native soybean rhizobia were isolated from farms without any history of inoculation, and characterized in terms of phylogeny, distribution and symbiotic effectiveness with the ultimate aim of developing inoculants for increased soybean yields in Mozambique.

## Materials and methods

### Soil analysis, bacterial isolation and characterization

Soybean nodules were sampled from TGx and non-TGx soybean varieties grown in smallholder farmers’ fields in different provinces of Mozambique (Table 1). Healthy green soybean plants were dug out using a shovel and carried in paper bags to the laboratory. The rhizosphere soils were stored at 4 °C prior to nutrient analysis at the Department of Agriculture, Soil, Water and Plant Tissue Laboratory, Institute of Plant Production, Western Cape, South Africa. The climatic information and soil nutrient analysis of the sampling sites are shown in Table 1. Nodules harvested from each field, were surface-sterilized by rinsing in 95% ethanol, submerged in 3% NaOCl (commercial bleach) for 30 s, and rinsed six times with distilled water [47]. The nodules were aseptically dissected, the central portion squashed, and the nodule suspension streaked onto yeast mannitol agar (YMA) plates, and incubated at 28 °C. The plates were observed for bacterial colonies up to 20 d.

**Table 1:** Locations, rainfall and soil chemical properties of the farming areas sampled in Mozambique.

Province Mozambique	Sampling sites	Rainfall (mm)	pH (KCL)	C (%)	N (%)	P (mg/kg)	K (mg/kg)	Mn (mg/kg)	Fe (mg/kg)
Tete	Ntengo	1337	4.4	1.4	0.09	5	56	169	97
	Ntengo		4.4	0.9	0.07	4	85	131	69
	Angonia	1081	4.4	1.2	0.08	9	91	149	110
	Chiphole		4.9	2.0	11.80	102	140	148	216

Zambezia	Mutequelesse	1800–2000	5.4	1.4	0.10	17	190	380	139
	Mutequelesse		5.5	0.9	0.07	88	128	164	165
	Mutequelesse		5.9	1.3	0.07	182	292	62	156
	Mutequelesse		5.7	1.6	0.11	96	137	50	108
	Ruace	2000–2500	5.1	1.6	0.11	27	399	253	175
	Ruace		5.2	2.2	0.13	76	493	382	167
	Ruace		5.2	2.6	0.15	150	170	175	381
	Ruace		5.7	1.4	0.12	48	76	296	187
	Serra	1500–2000	5.5	1.3	0.07	90	147	207	117
	Serra		7.3	1.3	0.11	389	662	269	216
	Magige		5.2	1.1	0.07	41	293	105	98
	Tetete		5.6	0.9	0.05	76	78	57	43

Manica	Sussudenga	901–1200	6.5	1.4	4.40	2	105	–	–

Nampula	Muriase	800–1200	5.4	1.5	0.12	12	110	295	120

### Authentication and measuring strain symbiotic efficiency

Seeds of soybean variety TGx1908-8F were surface-sterilized with 2% sodium hypochlorite (NaOCl) for 5 min, 70% alcohol for 1 min and thoroughly rinsed five times with sterile water. The seeds were aseptically planted in sterilised plastic pots containing sterile sand inside a laminar flow chamber. To prepare isolate cultures for inoculating the sterile-grown seedlings, one loopful of the bacterial culture was transferred to 6 ml yeast mannitol broth, incubated at 28 °C on a rotary shaker (65 cycles per minute) for seven days. Five days after planting (DAP), the seedlings were inoculated with 2 ml suspension of the bacterial culture (∼ 10^7^–10^8^ cells/ml) [43]. The pots with seedlings were transferred to the glasshouse. Three replicate plants were grown per rhizobial isolate, and Dilworth’s N-free nutrient solution was used to irrigate the plants [6]. Uninoculated and 5 mM NO_3_^−^-fed plants were included as controls. The plants were harvested at 45 DAP for assessing growth and nodulation.

The authenticated (nodule-forming) isolates were tested for acid and alkali production using bromothymol blue (25 μg/ml), as well as for bacterial colony size, shape, texture and number of days to grow and were further characterized genetically.

### Genomic DNA extraction and PCR-amplification of 16S rRNA gene fragment

Bacterial genomic DNA was extracted from authenticated rhizobial isolates using GenElut bacterial DNA isolation Kit (Sigma–Aldrich, USA) according to the manufacturer’s instructions. PCR amplification of the 16S rRNA of bacterial genome was carried out with 1 μl (40–60 ng/ul) DNA in a 25 μl reaction volume with 3 μl (5X) My Taq PCR buffer containing MgCl_2_ and dNTPs, 5U Taq polymerase (Bioline, USA), 10 pM of each primer, and double-distilled sterile water in a Thermal cycler (T100 Bio-Rad, USA). The primers and temperature profiles are described in Table S1. The size of the amplified products was estimated by gel electrophoresis on a 1.5% agarose gel stained with 1 μg ml^−1^ ethidium bromide using a standard DNA marker (GeneDirex, 1kbp and 100 bp ladder), and photographed using a gel documentation system (GeldocTm XR+, Bio-RAD, USA).

### Restriction Fragment Length Polymorphism (RFLP) analysis of the PCR-amplified 16S rRNA gene

The amplified 16S rRNA-PCR products were digested with three fast digest four base-cutting restriction endonucleases (*Hpa*II, *Hind*III and *Msp*I) separately following the instructions of the manufacturer (Thermo Scientific Lithuania, EU). The digested DNA fragments were separated by horizontal gel electrophoresis on a 3% agarose gel in Tris acetic acid EDTA (1X TAE) buffer at 85 V for 2.5 h, and photographed.

The RFLP groups were determined using the restriction fragment patterns generated by the different test restriction endonucleases. The relationship between isolates was established using data from the restriction enzymes that differentiated the isolates. A binary scoring system (1 for presence, and 0 for absence of a homologous band) was used to generate an input matrix for construction of a dendrogram using the unweighted pair-group method with the arithmetic mean algorithm (UPGMA) by means of NTSYS-Pc 2.1 software [39].

### Multilocus (16S rRNA, *glnII*, *gyrB, nifH,* and *nodC*) sequence and phylogenetic analyses

Representative isolates were selected from each Cluster based on the 16S rRNA PCR-RFLP analysis for subsequent sequence analysis of 16S rRNA, *gyrB*, *glnII* and symbiotic *nifH* and *nodC* genes. The PCR amplification of *gln*II*, gyr*B, *nif*H and *nodC* genes of the rhizobial genome was carried out as described above for 16S rRNA PCR amplification. The primers used and thermal cycle conditions are listed in Table S1. The PCR-amplified products of 16S rRNA, *glnII, gyrB and* symbiotic *nifH* and *nodC* genes were purified by Favour/Prep PCR purification kit (FAVORGEN, Sigma, USA), and sequenced (Macrogen, Netherlands). The ribosomal sequences were screened for chimeric sequences using UCHIME [11]. The quality of all nucleotide sequences was checked using BioEdit 7.0.0 software [16], and BLAST_n_ programme was used in the GenBank database (http://www.ncbi.nlm.nih.gov/) to identify closely related species. Nucleotide sequences were submitted to NCBI GenBank to get accession numbers (Table S2). Reference type sequences were obtained from NCBI GenBank to align with sequences of the test isolates using MUSCLE [10] for the construction of phylogenetic tree by means of MEGA 6.0 program [45]. Phylogenetic trees were generated by Kimura 2-parameter method and evolutionary history was inferred using Neighbor-Joining method with 1000 bootstrap support [13], [23], [40]. Nucleotide information was recorded from conserved, variable, parsimony-informative, and singleton regions using consensus sequences.

### Symbiotic data analysis

The symbiotic data (nodule number, nodule dry weight, plant dry weight) were subjected to a One-Way ANOVA using STATISTICA (Sta Soft Inc., Tulsa, OK, USA, 2012) [44] to test for significant differences, and means separated using Duncan’s multiple range test (DMRT) at P ≤ 0.05

### Factors responsible for rhizobial distribution in Mozambican soils

Constrained correspondence analysis (CCA) was performed to determine the correlation between environmental factors and 16S rRNA-RFLP data of the rhizobial communities. The combination of explanatory variables was determined to describe the most influential variables by conducting an ANOVA permutation test with 999 permutations in a reduced model (P < 0.05). The analysis was done by means of R (version 2.15.3) using the package vegan [32].

## Results

### Isolate characterization and symbiotic efficacy

Seventy pure bacterial isolates from soybean nodules were tested for their ability to form root nodules on soybean (the homologous host) in fulfilment of Koch’s postulates. About 61% of the isolates (43 out of 70) induced effective root nodules on the host plant under microbiologically-strict glasshouse conditions. Their effectiveness was evidenced by the pink red internal colouration of the nodules, as well as the dark green colour of leaves when compared to the whitish internal colour of ineffective nodules and the yellow leaves of plants inoculated with ineffective isolates. Symbiotic response of the 43 tested isolates with soybean variety TGx1908-8F revealed significantly marked differences in nodule number (*P* *≤* 0.001), nodule dry weight (*P* *≤* 0.01) and plant dry weight (*P* *≤* 0.001). The number of nodules formed per plant ranged from 2 for isolates TUTMJM6A and TUTMCJ7B, to 52 for TUTMFJ4. Isolates TUTM19373A, TUTMJM5, TUTMIITA5A, TUTLBC2B, TUTRAH5B2, TUTRJN3A1, TUTMIITA5A1 and TUTMCJ5B1 were highly effective, and recorded greater nodule number and plant biomass relative to NO_3_-fed plants (Table 2).

**Table 2:** Nodulation and symbiotic efficacy of forty three (43) native soybean bacteria isolates carried out under glasshouse conditions, and sampled at 45 days after planting.

Isolate	Location	Nodule number	Dry matter yield (g plant^−1^)		
			Nodule	Shoots + roots	Whole-plant
TUTNFM2A1	Ntengo	34 ± 3.8c-h	0.1 ± 0.0d	1.1 ± 0.2e-k	1.2 ± 0.2d-q
TUTMFJ4	Mutequelesse	52 ± 5.0a	0.3 ± 0.1bc	1.4 ± 0.1a-f	1.7 ± 0.2b-i
TUTRAH8A	Ruace	30 ± 5.1c-k	0.4 ± 0.0ab	1.0 ± 0.2f-l	1.4 ± 0.2d-n
TUTRAB5B1	Ruace	25 ± 1.2e-n	0.2 ± 0.0c	0.9 ± 0.1g-l	1.1 ± 0.1e-q
TUTMIITA5A2	Mutequelesse	34 ± 2.7c-h	0.1 ± 0.0d	1.4 ± 0.1a-f	1.6 ± 0.2b-k
TUTMFJ3B	Mutequelesse	23 ± 1.7g-n	0.1 ± 0.0de	1.0 ± 0.1f-l	1.1 ± 0.1f-q
TUTMFJ2B	Mutequelesse	33 ± 0.6c-i	0.1 ± 0.0de	1.6 ± 0.1abc	1.7 ± 0.1a-h
TUTMJM6A	Magige	2 ± 0.3s-t	0.0 ± 0.0e	1.0 ± 0.1f-l	1.0 ± 0.1f-q
TUTMIITA4A	Mutequelesse	5 ± 0.0t	0.0 ± 0.0e	0.8 ± 0.0jkl	0.8 ± 0.0h-q
TUTMFJ1A1	Mutequelesse	22 ± 3.8h-o	0.1 ± 0.0de	0.9 ± 0.1h-l	1.0 ± 0.1f-q
TUTM19373A	Muriase	30 ± 6.4c-k	0.3 ± 0.1bc	1.9 ± 0.3a	2.2 ± 0.3a
TUTM19043A	Muriase	7 ± 1.2o-t	0.1 ± 0.0de	0.9 ± 0.2h-l	0.9 ± 0.2e-q
TUTRAH5B2	Ruace	42 ± 4.6a-d	0.2 ± 0.0c	1.7 ± 0.1ab	1.8 ± 0.1a-f
TUTRSRH8B	Ruace	27 ± 0.6d-l	0.1 ± 0.0d	1.2 ± 0.1c-k	1.3 ± 0.1g-p
TUTRJN5A	Ruace	39 ± 18.2a-e	0.1 ± 0.1d	1.4 ± 0.3a-g	1.4 ± 0.4d-m
TUTNFM1A	Ntengo	15 ± 1.2k-s	0.1 ± 0.0de	0.8 ± 0.1i-l	0.9 ± 0.1f-q
TUTLBC1B	Angonia	8 ± 1.0o-t	0.1 ± 0.0de	1.5 ± 0.0a-f	1.5 ± 0.1c-l
TUTMCJ9A	Mutequelesse	12 ± 1.5m-t	0.2 ± 0.1c	0.8 ± 0.1i-l	1.0 ± 0.2f-q
TUTMCJ5B	Mutequelesse	26 ± 4.5e-m	0.1 ± 0.1de	0.8 ± 0.1j-l	0.8 ± 0.2g-q
TUTMJM5	Magige	16 ± 0.7k-s	0.4 ± 0.1a	1.6 ± 2.0b-k	2.0 ± 0.2ab
TUTDAIAP3B	Angonia	44 ± 6.4abc	0.2 ± 0.1 cd	0.8 ± 0.1i-l	1.0 ± 0.2e-q
TUTMCJ4B1	Mutequelesse	20 ± 0.6h-p	0.1 ± 0.0de	0.9 ± 0.1g-l	1.0 ± 0.1f-q
TUTDAIAP1A	Angonia	51 ± 7.9j-q	0.3 ± 0.1bc	1.2 ± 0.1f-l	1.5 ± 0.2c-l
TUTDAIAP2A1	Angonia	34 ± 1.4abc	0.2 ± 0.0 cd	1.5 ± 0.1b-i	1.7 ± 0.1b-h
TUTMIITA5A	Mutequelesse	51 ± 7.9ab	0.4 ± 0.0ab	1.6 ± 0.1b-k	2.0 ± 0.1ab
TUTRJN3A1	Ruace	16 ± 1.5k-p	0.2 ± 0.0 cd	1.7 ± 0.1a-f	1.9 ± 0.2a-d
TUTNSN2A	Ntengo	11 ± 2.4m-t	0.1 ± 0.0def	0.9 ± 0.0abcd	1.0 ± 0.0e-q
TUTLBC2B	Angonia	41 ± 2.9a-d	0.4 ± 0.0a	1.6 ± 0.1a-f	2.0 ± 0.2a-d
TUTDAIAP8B	Angonia	20 ± 0.8h-p	0.0 ± 0.0de	0.6 ± 0.1g-l	0.6 ± 0.1h-q
TUTMCJ10B	Mutequelesse	6 ± 1.3p-t	0.2 ± 0.0 cd	1.0 ± 0.1a-f	1.2 ± 0.1d-l
TUTRLR4B2	Ruace	15 ± 2.0n-t	0.2 ± 0.0c	1.5 ± 0.1l	1.7 ± 0.1b-h
TUTSFD1A	Serra	22 ± 0.0rst	0.2 ± 0.0 cd	1.2 ± 0.1f-l	1.4 ± 0.1d-m
TUTNFM3B	Ntengo	31 ± 1.8c-j	0.2 ± 0.0 cd	1.6 ± 0.1b-k	1.7 ± 0.1b-h
TUTNSN3B	Ntengo	41 ± 0.5a-d	0.1 ± 0.0d	0.9 ± 0.2c-k	1.0 ± 0.2e-n
TUTMCJ4B	Mutequelesse	37 ± 2.6b-g	0.1 ± 0.0d	0.8 ± 0.1abcd	0.8 ± 0.2f-q
TUTMIITA5A1	Mutequelesse	28 ± 2.0d-l	0.3 ± 0.0bcd	1.5 ± 0.1a-f	1.8 ± 0.1bcd
TUTMCJ5B1	Muteuelesse	14 ± 1.5l-t	0.2 ± 0.1c	1.7 ± 0.1jkl	1.9 ± 0.2bcd
TUTRSRH9A	Ruace	17 ± 0.3j-r	0.3 ± 0.0bcd	1.2 ± 0.2a-e	1.5 ± 0.2a-g
TUTRLR3B	Ruace	38 ± 12.3a-f	0.3 ± 0.2b	1.3 ± 0.2d-k	1.6 ± 0.2b-k
TUTRAB2B	Ruace	23 ± 1.8f-n	0.1 ± 0.0def	1.0 ± 0.1c-k	1.1 ± 0.1f-p
TUTNSN3B1	Ntengo	44 ± 6.0abc	0.3 ± 0.0b	1.2 ± 0.3b-h	1.5 ± 0.3c-l
TUTMFJ3BG	Mutequelesse	19 ± 0.8i-p	0.0 ± 0.0e	1.5 ± 0.1a-d	1.5 ± 0.1c-l
TUTMCJ7B	Mutequelesse	2 ± 0.3st	0,0 ± 0.0e	0.7 ± 0.1b-j	0.7 ± 0.1g-q
5 mM KNO_3_	na	0 ± 0.0t	0.0 ± 0.0f	1.5 ± 0.0a-e	1.5 ± 0.0
Control	na	0 ± 0.0t	0.0 ± 0.0f	0.1 ± 0.0kl	0.1 ± 0.0d-q
F-statistics	na	11.4***	19.0**	4.6***	6.02***

^Values (Means ± SE) with dissimilar letters in a column are significant at **P ≤ 0.01 and ***P ≤ 0.001.^

The 43 effective isolates obtained after authentication varied in shape, with 79% forming round colonies and 21% dome-shaped colonies. The majority of the isolates had a colony diameter of 2–3 mm. About 80% of the isolates were opaque, and 20% translucent. One isolate was gummy, while the remaining were mostly smooth and non-gummy. A bromothymol blue (BTB) test showed that 80% of the isolates tested were alkali-producing (turning BTB blue), while 20% were acid-producing (turning BTB yellow) (data not shown).

### Restriction Fragment Length Polymorphism (RFLP) analysis of the PCR-amplified 16S rRNA gene

PCR amplification using the 16S rRNA primers produced 1500 bp DNA fragments from the genome of the bacterial isolates. Digesting the 16S rRNA fragment with three different restriction enzymes (i.e. *Hae*III, *Hpa*II and *Msp*I) resulted in clear polymorphic RFLP band patterns. The RFLP patterns produced by each of the restriction enzymes are shown in Table S3.

**Fig. 1:**
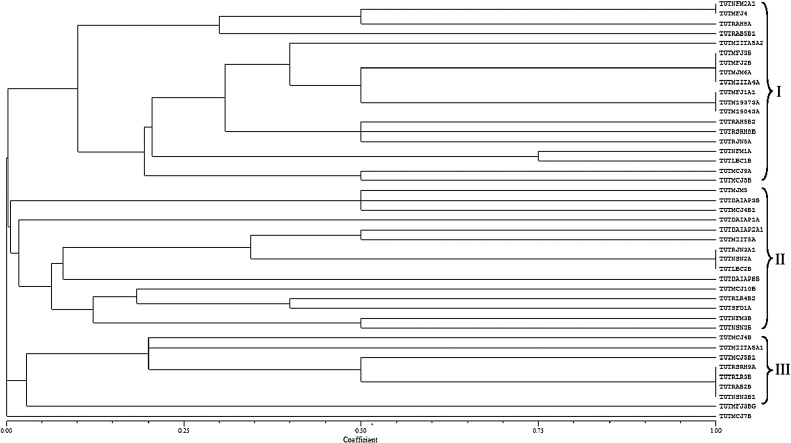
Dendrogram constructed from the combined 16S rRNA-RFLP banding patterns digested with MspI, HpaII and HaeIII restriction endonucleases.

Digestion of 16S rRNA genomic region of the soybean isolate with restriction endonucleases showed *Msp*I and *Hpa*II had the most polymorphic (18 patterns) activity compared to *Hae*III (14 patterns). The RFLP banding patterns generated from the three restriction endonucleases were used to construct a dendrogram by applying the UPGMA method. The analysis resulted in three major clusters (Fig. 1). Cluster I comprised 19 isolates, Cluster II 15 isolates, and Cluster III had 8 isolates. A total of 32 combined 16S rRNA-RFLP patterns were obtained. All the isolates grouped together with a 0.0–1.0 Jaccard’s similarity co-efficient. One isolate (TUTMCJ7B) did not cluster with any of the other test isolates and showed 0.0 Jaccard’s similarity co-efficient relative to the others. Cluster I, which comprised 19 isolates, grouped together with a similarity co-efficient of 0.10, while with Clusters II and III each was grouped with a similarity co-efficient of 0.01 and 0.02, respectively. Clustering of isolates was not based on location and soybean varieties (Fig. 1).

**Phylogenetic analysis of 16S rRNA, *gyr*B and *gln*II genes**

**Fig. 2:**
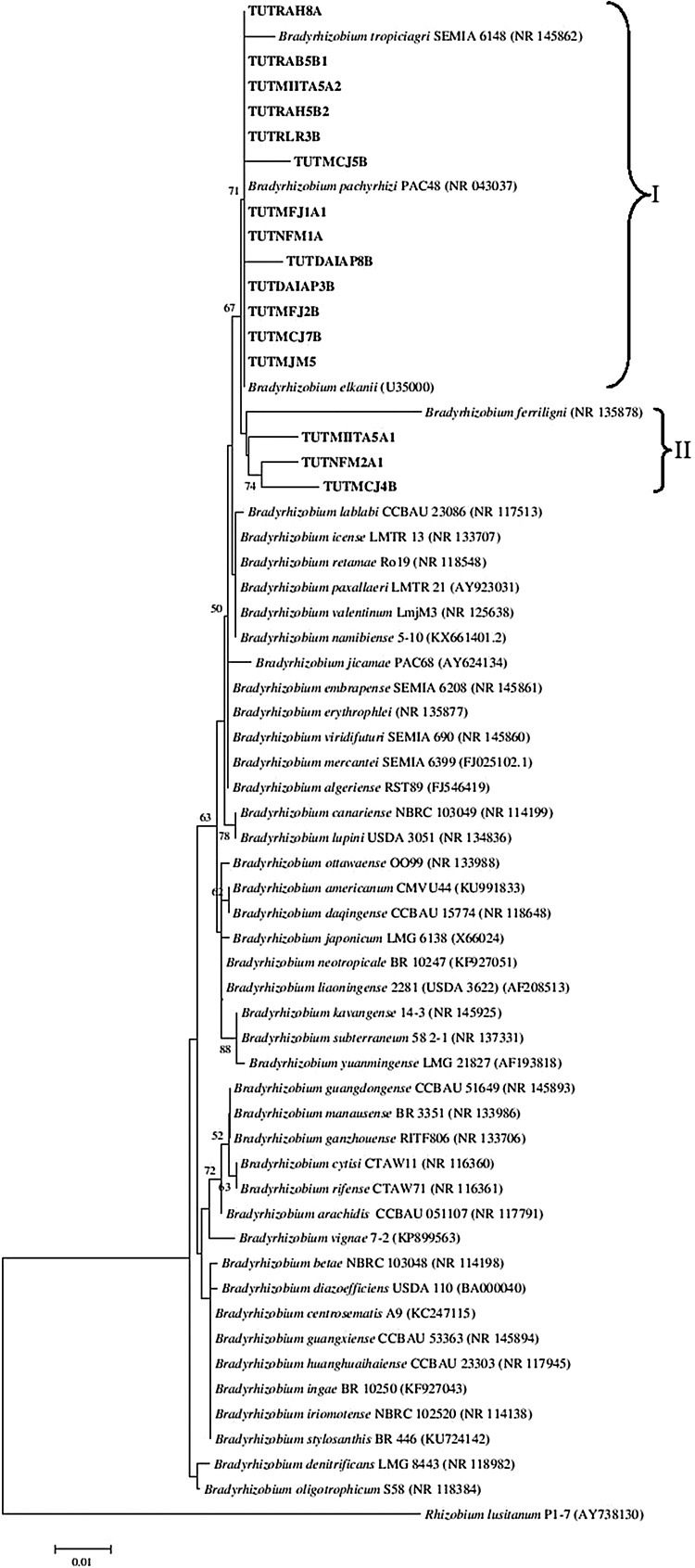
Phylogenetic tree based on 16S rRNA sequences generated by Neighbour-joining algorithm. Bootstrap values (1000 replicates) are indicated above the branches.

The BLAST_n_ analysis of 16S rRNA sequences of representative soybean isolates selected from each cluster of 16S rRNA-RFLP identified the isolates as *Bradyrhizobium*. The test isolates occupied two Clusters (I–II). Phylogenetically, 11 isolates (TUTMCJ7B, TUTRAH5B2, TUTMFJ1A1, TUTMFJ2B, TUTMIITA5A2, TUTMJM5, TUTNFM1A, TUTRLR3B, TUTDAIAP3B, TUTRAB5B1, and TUTRAH8A) closely grouped with reference type strains *B. elkanii* and *B. pachyrhizi* in Cluster I with 71% bootstrap support (Fig. 2) and 100% sequence identity. Isolates TUTNFM2A1, TUTMIITA5A1, and TUTMCJ4B comprised Cluster II and showed 98.3 − 98.9% sequence identity with the type strain of *B. elkanii* and *B. pachyrhizi*. Isolates TUTDAIAP8B and TUTMCJ5B formed outgroups of Cluster I and showed 99.0 and 99.1% sequence identity, respectively, with *B. elkanii* (Fig. 2).

PCR-amplification of *glnII* and *gyrB* housekeeping genes resulted in 700 bp single band of each isolate. The *gln*II and *gyrB* housekeeping genes respectively encode glutamine synthetase II, and DNA gyrase B-subunit. The sequence analysis of these housekeeping genes showed that the highest conserved (62.70%) and parsimony-informative (26.46%) was found in *glnII* consensus sequences while *gyrB* sequences had the highest variable (63.49%) and singleton (18.15%) positions (Table S4).

**Fig. 3:**
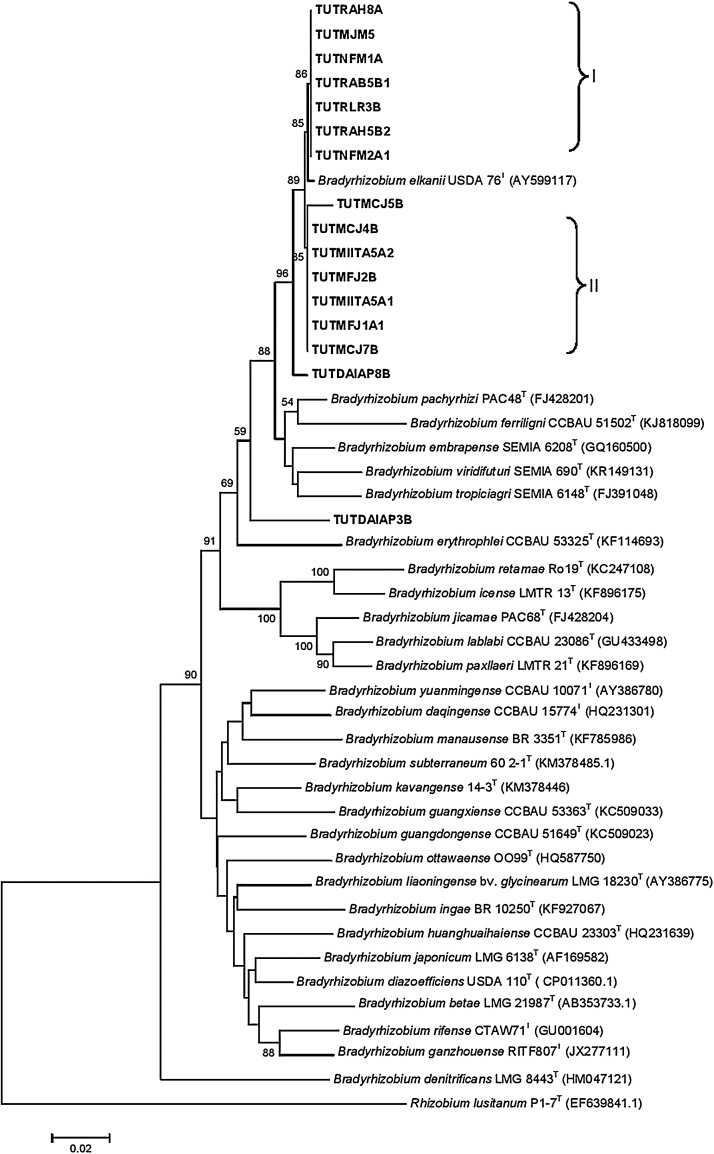
Concatenated phylogenetic tree based on *glnII* + *gyrB* sequences generated by Neighbour-joining algorithm. Bootstrap values (1000 replicates) are indicated above the branches.

The phylogenetic analysis of both *glnII* and *gyrB* housekeeping genes grouped the test isolates into two clusters (Figs. S1 & S2). The isolates of Cluster I were closely related to *B. elkanii* with 100 and 99% bootstrap support in their respective phylogenies and high sequence identity of 98.9-100%. Isolate TUTMCJ5B formed an outgroup of Cluster I in the *glnII* phylogeny and showed 96.8% sequence identity with *B. elkanii* but grouped in Cluster I in the *gyrB* phylogeny with 100% sequence identity. Isolate TUTDAIAP3B formed Cluster II and showed proximal relation with *B. arachidis* with 96% sequence identity while it grouped with TUTDAIAP8B in Cluster II in the *gyrB* phylogeny and showed 98.5% sequence identity with *B. elkanii*. The aligned sequences of *glnII +gyrB* were combined and 918 analysed sites were used to construct a concatenated tree to refine the positions of the test isolates (Fig. 3). The concatenated tree was based on 544 conserved, 368 variable, and 227 parsimony — informative sites (Table S4).

The phylogenetic tree constructed from the concatenated genes grouped the isolates into two major clusters (I–II) with *B. elkanii* type strain (Fig. 3) with sequence identity of 99.5–99.6%. However, test isolates TUTDAIAP8B and TUTMCJ5B were out-grouped and showed 98.6% sequence identity with *B. elkanii*. Isolate TUTDAIAP3B stood alone without any reference type strains and showed proximal relation with *B. elkanii* with 95.1% sequence identity (Fig. 3)

**Phylogenetic analysis based on *nif*H and *nodC* gene sequences**

To determine the presence and diversity of the N_2_-fixing genes (*nif*H and *nodC*) among the soybean rhizobia, test isolates were used to successfully amplify 600 bp and 300 bp of the *nif*H and *nodC* genome regions, respectively, and sequenced. From incompatibility of primer pairs, only 11 isolates were able to yield *nodC* amplifications. Due to the presence of small length of *nifH* reference sequences, 274 nucleotide length sequences were used for phylogenetic analysis. The analysed *nifH* 274 nucleotide sequences comprised 165 conserved, 109 variables and 86 parsimony informative sites (Table S4). All the test isolates in the *nifH* phylogram grouped into two clusters (Cluster I–II).

**Fig. 4:**
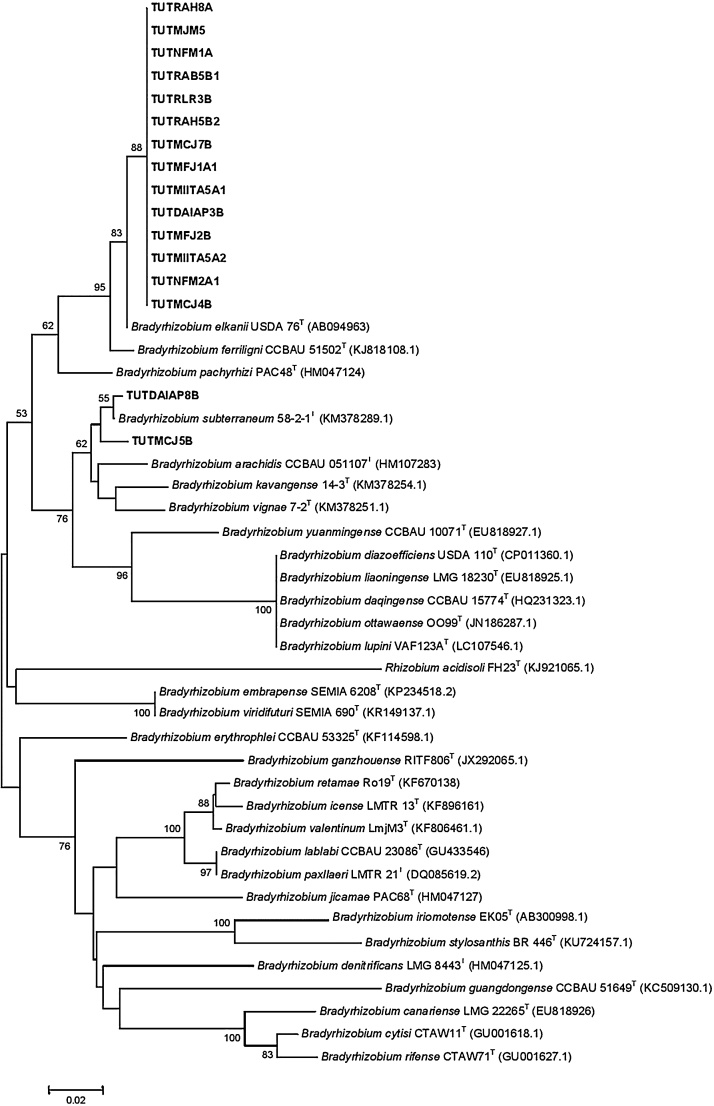
Phylogenetic tree based on *nifH* sequences generated by Neighbour-joining algorithm. Bootstrap values (1000 replicates) are indicated above the branches.

**Fig. 5:**
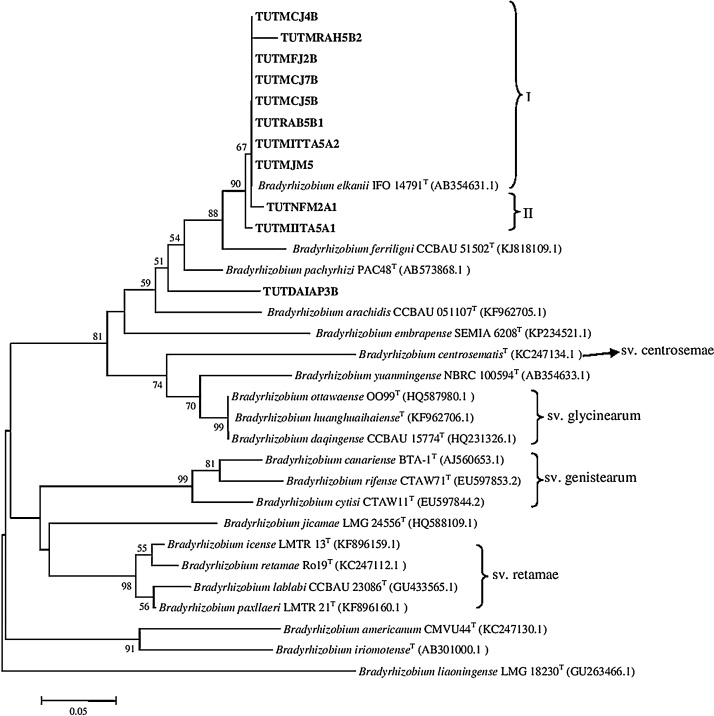
Phylogenetic tree based on *nodC* sequences generated by Neighbour-joining algorithm. Bootstrap values (1000 replicates) are indicated above the branches.

Fourteen isolates in Cluster I were closely related to *B. elkanii* with 99.2% sequence identity and 88% bootstrap support. But isolates TUTDAIAP8B and TUTMCJ5B in Cluster II were closely aligned with *B. subterraneum* with 99.6 and 98.1% sequence identity, respectively (Fig. 4). The test isolates in the *nodC* phylogeny were placed in two Clusters. In Cluster I, the isolates were grouped with *B. elkanii* with 99.4-100% sequence identity. Isolates TUTMIITA5A1 and TUTNFM2A1 showed proximal relation with 90.8 and 98.4% sequence identity, respectively, with *B. elkanii* while isolate TUTDAIAP3B stood alone in the phylogram (Fig. 5).

### Factors responsible for rhizobial distribution in Mozambican soils

**Fig. 6:**
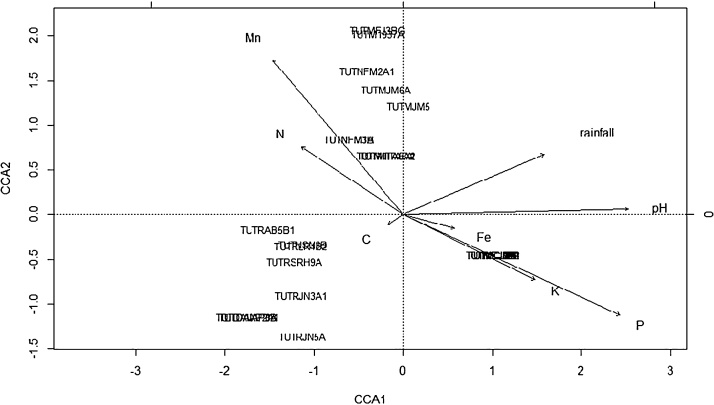
CCA ordination diagram showing the relationship between rhizobial communities and environmental variables.

The physico-chemical properties of soil (Mn, Fe, C, K, P, N and pH) and rainfall data were combined with 16S rRNA-RFLP score to determine the distribution of soybean-nodulating isolates in Mozambique. The results of CCA analysis showed that 70% of the variation were explained, while 30% were unexplained. The CCA analysis also showed that soil pH was the most important environmental variable influencing the distribution of soybean rhizobia (Fig. 6), followed by plant-available P and Mn in soil. According to the angles and lengths of the arrows, it was observed that pH and available P were strongly positively correlated (90%) with the distribution of soybean rhizobia at Mutequelesse (TUTMCJ9A, TUTMCJ5B, TUTMCJ4B1, TUTMCJ4B, TUTMCJ10B and TUTMCJ7B). However, pH and rainfall were negatively correlated with the distribution of the soybean isolates at Ruace (TUTRAB5B1, TUTNSN3B, TUTRLR3B, TUTRSRH9A, TUTRJN3A1 and TUTRJN5A) and Angonia (TUTDAIAP8B and TUTDAIAP3B, TUTDAIAP1A and TUTDAIAP2A1). The distribution of soybean isolates TUTMFJ3BG (from Mutequelesse), TUTNFM2A1, TUTNFM3B (from Ntengo), TUTM19373A, TUTMIITA5A2, TUTMIITA5A and TUTMIITA5A1 (from Muriase) were moderately influenced by Mn (52%). But soil Fe and C had a low influence on the distribution of the soybean isolates as shown by their short arrows. The large angle between Mn and pH, as well as Mn and P indicates that they had independent effects on the distribution of the soybean isolates.

## Discussion

The genetic diversity of native rhizobia nodulating soybean in Mozambique has little been studied. However, the renewed government interest in promoting soybean production in Mozambique necessitates the creation of new knowledge for increasing the grain yield of this legume. Understanding the genetic diversity of soybean rhizobia is important for discovering new genera, species and strains for developing new technologies, including the production of inoculants, for increased soybean yield. To explore the genetic diversity of native soybean rhizobia in Mozambican soils, nodules were sampled from farmers’ fields that had no previous history of rhizobia inoculation, and analysed genetically.

Although analysis of genomic DNA revealed a high diversity in the 16S rRNA-RFLP results as evidenced by the different banding patterns following digestion with the three enzymes, the MLSA data showed alignment of the isolates with *B. elkanii* species. These results (RFLP and phylogenies) suggest that, there is an intra-species diversity within the *B. elkanii* group of native rhizobia nodulating soybean in Mozambique. However, the 16S rRNA sequences grouped the isolates with *B. elkanii, B. pachyrhizi, B. tropiciagri* and *B. ferriligni.* This suggests that sequencing the 16S rRNA gene alone is insufficient for differentiating *Bradyrhizobium* species [3], [9], [38], and indicates that rhizobial 16S rRNA gene phylogeny may not always accurately reflect prokaryotic phylogeny. Housekeeping genes are highly conserved among bacteria of the order Rhizobiales, as they encode for important proteins [52] and have therefore been widely used in studies of phylogenetic reconstruction of the order Rhizobiales [27], [37]. As a result, two housekeeping genes (*gyr*B, *gln*II) were sequenced and analysed to support the 16S rRNA data. The phylogenetic analysis of individual housekeeping genes also suggested that most of the isolates were highly aligned with *B. elkanii*. However, the phylogenetic positions of isolates TUTDAIAP3B, TUTDAIAP8B and TUTMCJ5B were not stable in single gene phylogenies, and this could suggest recombination, migration or horizontal gene transfer [8], [36].

To fine-tune the individual housekeeping gene analysis, a concatenated phylogeny was done, and the results further confirmed that the isolates nodulating soybean in Mozambique were species of *Bradyrhizobium,* closely related to *B. elkanii*. Except isolates TUTMCJ5B and TUTDAIAP8B, there was phylogenetic congruence between the individual and concatenated housekeeping genes for all the isolates. The high similarity of the isolates (99.5–99.6%) with species belonging to the *B. elkanii* lineage indicates their close identity to *B. elkanii* [21], which suggests that the *B. elkanii* group may be the dominant microsymbiont of soybean in Mozambican soils.

Some studies have shown a relationship between soil environment and nodule occupancy. For example, *B japonicum* and *B. elkanii* were commonly isolated from acidic and neutral soils, while high pH decreased or completely eliminated their occupancy of root nodules in such soils [26], [53]. In this study, the soybean strains obtained were mostly from acidic to neutral soils with pH ranging from 4.4 to 7.0. Previous studies have found *B. elkanii* to be the dominant microsymbiont nodulating soybean varieties in Kenya [18], Myanmar [41], as well as other parts of Africa [1], [20], tropical and subtropical Asia [26], North America [5] and South America [4]. While *B. japonicum* and *B. elkanii* species are the known bacterial symbionts of soybean found across many geographic and climatic regions, worldwide *B. liaoningense*, *B. yuanmingense* and *Bradyrhizobium canariense* have been isolated from root nodules under limited climatic conditions [50], [51]. Recently, Naamala et al. [29] showed that *Bradyrhizobium diazoefficiens* and an unknown group of novel *Bradyrhizobium* species are the dominant bacteria nodulating soybean in South African soils.

The important nodulation and nitrogen fixation genes *nodC* and *nifH,* respectively, are situated on interchangeable elements such as symbiotic islands and plasmids, and play an important role in the outcome of the legume-*Rhizobium* symbiosis. From the results of *nif*H gene sequence analysis, the majority of soybean isolates were closely related to *B. elkanii*. However, some of the isolates also had *nif*H genes that were closely related to *B. subterraneum* and *B. arachidis*, thus suggesting that the *nif*H gene of these isolates (TUTDAIAP8B and TUTMCJ5B) probably have different origin compared to known bradyrhizobial strains. Even in 16SrRNA and concatenated gene sequence analyses, isolates TUTDAIAP8B and TUTMCJ5B showed low sequence identity with known *B. elkanii* type strains. The inconsistency of *nodC* with core gene phylogenies for isolates TUTMIITA5A1, TUTRAH5B2 and TUTNFM2A1 suggested differences in evolutionary history of both chromosomal and symbiotic genes [8].

Glasshouse nodulation assays revealed variable induction of root nodules and symbiotic effectiveness among the bacterial isolates. The nodules formed differed in shape and size, with smaller nodules ranging from 0.1 to 0.2 mm, medium-sized nodules ranging from 0.3 to 0.4 mm in diameter relative to 0.5–0.6 mm in diameter for bigger nodules. Isolates that induced bigger nodules generally produced fewer nodule numbers, while those that induce smaller nodules produce greater nodule numbers. This could explain why some isolates such as TUTM19043A which recorded only 7 nodules, showed nodule dry weight similar to TUTRSRH8B that produced more nodules. Some isolates which recorded higher nodule number (e.g. isolate TUTM19373A), also recorded a higher biomass (see Table 2). By contrast, isolate TUTMJM5 recorded lower nodule number but showed high symbiotic effectiveness comparable to isolate TUTM19373A. This finding indicates that nodule number alone is not a good measure of symbiotic effectiveness of a strain. Isolates TUTM19373A, TUTMJM5, TUTRJN3A1, TUTLBC2B, TUTMCJ5B1 TUTMIITA5A1, TUTMIITA5A, TUTRAH5B2, TUTMFJ4, TUTMFJ2B TUTDAIAP2A1, TUTRLR4B2 and TUTNFM3B were highly effective and promoted plant growth that was greater than that of 5 mM NO^3−^-fed plants. These isolates occurred in Clusters I and II in the RFLP dendrogram, and representative isolates used for the 16S and *nif*H analysis clustered together with *B. elkanii* in both analyses. However, isolates TUTDAIAP8B (which showed poor symbiotic ability) and TUTRAB5B1 (which exhibited moderate effectiveness), occurred in Clusters I and II of the RFLP dendrogram but separated from the remaining isolates that aligned with *B.elkanii* and showed distant relation with *B. ferriligni*. However, TUTRAB5B1 had nitrogen-fixing genes similar to *B. elkanii*, while TUTDAIAP8B had *nif*H genes closely related to *B. subterraneum*. Isolates TUTNFM2A1 (moderately effective) and TUTMCJ4B (poorly effective) clustered together (Cluster III) in the 16S analysis, and showed close relatedness with *B. elkanii* in terms of their nitrogen-fixing genes.

Strains isolated from Ruace soils were particularly very interesting because they generally promoted greater whole-plant biomass. This observation was not surprising as earlier studies on quantification of N_2_ fixation at three locations in Mozambique found that soybean plants from Ruace were symbiotically superior to their counterparts from the other locations irrespective of inoculation [14].

In this study, constrained correspondence analysis (CCA) was done in order to better understand the influence of rainfall and soil characteristics on the biogeographic distribution of native rhizobia nodulating soybean in Mozambique. As found in other studies [25], [49], [53], the distribution of soybean rhizobia was markedly influenced by soil pH. In fact, soil pH has been reported to be a major determinant of the biogeography of *Bradyrhizobium* species [25], [26], followed by plant-available P and Mn in the soil. Elsewhere, soil pH, salinity and phosphate levels have also been identified as important parameters determining the biogeographic distribution of soybean rhizobia [17]. In conclusion, this study has identified *B. elkanii* as the major native rhizobial species nodulating soybean in Mozambican soils. Strain symbiotic effectiveness studies also suggest that some native rhizobia such as strains TUTM19373A, TUTMJM5, TUTMIITA5A and TUTLBC2B present in Mozambican soils have the potential for use as inoculants to increase soybean production. The study further revealed that soil pH, P and Mn were the most important factors determining the distribution of soybean rhizobia in Mozambique.

## References

[1] Abaidoo R.C., Keyser H.H., Singleton P.W., Borthakur D. (2002). Comparison of molecular and antibiotic resistance profile methods for the population analysis of *Bradyrhizobium* spp. (TGx) isolates that nodulate the new TGx soybean cultivars in Africa. J. Appl. Microbiol..

[2] Africa N. (2013). Soya production: the Mozambique experience. Putting nitrogen fixation to work for smallholder farmers in Africa.

[3] de Almeida Ribeiro P.R., dos Santos J.V., da Costa E.M., Lebbe L., Assis E.S., Louzada M.O., Guimarães A.A., Willems A., de Souza Moreira F.M. (2015). Symbiotic efficiency and genetic diversity of soybean bradyrhizobia in Brazilian soils. Agric. Ecosyst. Environ..

[4] Barcellos F.G., Menna P., da Silva Batista J.S., Hungria M. (2007). Evidence of horizontal transfer of symbiotic genes from a *Bradyrhizobium japonicum* inoculant strain to indigenous diazotrophs *Sinorhizobium* (*Ensifer*) *fredii* and *Bradyrhizobium elkanii* in a Brazilian Savannah soil. Appl. Environ. Microbiol..

[5] Berkum P., van, Fuhrmann J.J. (2001). Characterization of soybean bradyrhizobia for which serogroup affinities have not been identified. Can. J. Microbiol..

[6] Broughton W.J., Dilworth M.J. (1971). Control of leghaemoglobin synthesis in snake beans. Biochem. J..

[7] Champion R.A., Mathis J.N., Israel D.W., Hunt P.G. (1992). Response of soybean to inoculation with efficient and inefficient *Bradyrhizobium japonicum* variants. Crop Sci..

[8] Chidebe I.N., Jaiswal S.K., Dakora D. (2018). Distribution and phylogeny of microsymbionts associated with cowpea (*Vigna unguiculata*) nodulation in three agroecological regions of Mozambique. Appl. Environ. Microbiol..

[9] Delamuta J.R., Ribeiro R., Menna P., Bangel E., Hungria M. (2012). Multilocus sequence analysis (mlsa) of *Bradyrhizobium* strains: revealing high diversity of tropical diazotrophic bacteria. Braz. J. Microbiol..

[10] Edgar R.C. (2004). MUSCLE: multiple sequence alignment with high accuracy and high throughput. Nucleic Acids Res..

[11] Edgar R.C., Haas B.J., Clemente J.C., Quince C., Knight R. (2011). UCHIME improves sensitivity and speed of chimera detection. Bioinformatics.

[12] Estrada J.M. (2004). Regional overview of the soybean markets: challenges and opportunities for smallholder farmers in Southern Africa. ICRISAT, Patancheru.

[13] Felsenstein J. (1985). Confidence limits on phylogenies: an approach using the bootstrap. Evolution (N. Y.).

[14] Gyogluu C., Boahen S.K., Dakora F.D. (2016). Response of promiscuous-nodulating soybean (*Glycine max* L. Merr.) genotypes to *Bradyrhizobium* inoculation at three field sites in Mozambique. Symbiosis.

[15] Gyogluu C., Mohammed M., Jaiswal S.K., Kyei-boahen S., Dakora F.D. (2017). Assessing host range, symbiotic effectiveness, and photosynthetic rates induced by native soybean rhizobia isolated from Mozambican and South African soils. Symbiosis.

[16] Hall T. (2004). BioEdit version 7.0. 0. Distributed by the author. arxiv:/www.mbio.ncsu.edu/BioEdit/bioedit.html.

[17] Han L.L., Wang E.T., Han T.X., Liu J., Sui X.H., Chen W.F., Chen W.X. (2009). Unique community structure and biogeography of soybean rhizobia in the saline-alkaline soils of Xinjiang, China. Plant Soil.

[18] Herrmann L., Chotte J.-L., Thuita M., Lesueur D. (2014). Effects of cropping systems, maize residues application and N fertilization on promiscuous soybean yields and diversity of native rhizobia in Central Kenya. Pedobiologia (Jena).

[19] IITA (2016). Of grains and gains: realizing the potential of soybean in Mozambique. Gearing up for impact IITA 2015 Annual Report.

[20] Jaiswal S.K., Beyan S.M., Dakora F.D. (2016). Distribution, diversity and population composition of soybean-nodulating bradyrhizobia from different agro-climatic regions in Ethiopia. Biol. Fertil. Soils.

[21] Janda J.M., Abbott S.L. (2007). 16S rRNA gene sequencing for bacterial identification in the diagnostic laboratory: pluses, perils, and pitfalls. J. Clin. Microbiol..

[22] Jarvis B.D.W., Downer H.L., Young J.P.W. (1992). Phylogeny of fast-growing soybean-nodulating rhizobia supports synonymy of Sinorhizobium and *Rhizobium* and assignment to *Rhizobium fredii*. Int. J. Syst. Evol. Microbiol..

[23] Kimura M. (1980). J. Mol. Evol..

[24] Kueneman E.A., Root W.R., Dashiell K.E., Hohenberg J. (1984). Breeding soybeans for the tropics capable of nodulating effectively with indigenous *Rhizobium* spp. Plant Soil.

[25] Li Q.Q., Wang E.T., Zhang Y.Z., Zhang Y.M., Tian C.F., Sui X.H., Chen W.F., Chen W.X. (2011). Diversity and biogeography of rhizobia isolated from root nodules of *Glycine max* grown in Hebei Province, China. Microb. Ecol..

[26] Man C.X., Wang H., Chen W.F., Sui X.H., Wang E.T., Chen W.X. (2008). Diverse rhizobia associated with soybean grown in the subtropical and tropical regions of China. Plant Soil.

[27] Martens M., Dawyndt P., Coopman R., Gillis M., De Vos P., Willems A. (2008). Advantages of multilocus sequence analysis for taxonomic studies: a case study using 10 housekeeping genes in the genus *Ensifer* (including former *Sinorhizobium*). Int. J. Syst. Evol. Microbiol..

[28] Musiyiwa K., Mpepereki S., Giller K.E. (2005). Symbiotic effectiveness and host ranges of indigenous rhizobia nodulating promiscuous soyabean varieties in Zimbabwean soils. Soil Biol. Biochem..

[29] Naamala J., Jaiswal S.K., Dakora F.D. (2016). Microsymbiont diversity and phylogeny of native bradyrhizobia associated with soybean (*Glycine max* L. Merr.) nodulation in South African soils. Syst. Appl. Microbiol..

[30] Okereke G.U., Eaglesham A.R.J. (1993). Nodulation and nitrogen fixation by 79 promiscuous soybean genotypes in a soil in east Nigeria. Agron. Afr..

[31] Okogun J.A., Sanginga N. (2003). Can introduced and indigenous rhizobial strains compete for nodule formation by promiscuous soybean in the moist savanna agroecological zone of Nigeria?. Biol. Fertil. Soils.

[32] Oksanen, J., Blanchet, F.G., Kindt, R., Legendre, P., et al., 2016. vegan: community ecology package. R Packag. Version, 0–2.

[33] Osunde A.O., Gwam S., Bala A., Sanginga N., Okogun J.A. (2003). Responses to rhizobial inoculation by two promiscuous soybean cultivars in soils of the Southern Guinea savanna zone of Nigeria. Biol. Fertil. Soils.

[34] Pal U.R., Norman J.C. (1987). Differential response of soybean cultivars promiscuous to native rhizobia to split application of fertilizer N in Nigerian savanna. J. Agron. Crop Sci..

[35] Pule-Meulenberg F., Gyogluu C., Naab J., Dakora F.D. (2011). Symbiotic N nutrition, bradyrhizobial biodiversity and photosynthetic functioning of six inoculated promiscuous-nodulating soybean genotypes. J. Plant Physiol..

[36] Puozaa D.K., Jaiswal S.K., Dakora F.D. (2017). African origin of *Bradyrhizobium* populations nodulating Bambara groundnut (*Vigna subterranea* L. Verdc) in Ghanaian and South African soils. PloS one.

[37] Ribeiro R.A., Ormeño-Orrillo E., Dall’Agnol R.F., Graham P.H., Martinez-Romero E., Hungria M. (2013). Novel Rhizobium lineages isolated from root nodules of the common bean (*Phaseolus vulgaris* L.) in Andean and Mesoamerican areas. Res. Microbiol..

[38] Rivas R., Martens M., de Lajudie P., Willems A. (2009). Multilocus sequence analysis of the genus *Bradyrhizobium*. Syst. Appl. Microbiol..

[39] Rohlf F. (2009). NTSYSpc: numerical taxonomy system. ver. 2.21c.

[40] Saitou N., Nei M. (1987). The neighbor-joining method: a new method for reconstructing phylogenetic trees. Mol. Biol. Evol..

[41] Soe K.M., Yamakawa T., Hashimoto S., Sarr P. (2013). Phylogenetic diversity of indigenous soybean bradyrhizobia from different agro-climatic regions in Myanmar. Sci. Asia.

[42] Solidaridad (2014). Sustainable soy production: Technoserve Inc. and Solidaridad team up in Mozambique.

[43] Somasegaran P., Hoben H.J. (1994). Counting rhizobia by a plant infection method. Handbook for Rhizobia.

[44] StatSoft Inc (2011). STATISTICA (data analysis software system), version 10.

[45] Tamura K., Stecher G., Peterson D., Filipski A., Kumar S. (2013). MEGA6: molecular evolutionary genetics analysis version 6.0. Mol. Biol. Evol..

[46] Tan Z.-Y., Xu X.-D., Wang E.-T., Gao J.-L., Martinez-Romero E., Chen W.-X. (1997). Phylogenetic and genetic relationships of *Mesorhizobium tianshanense* and related rhizobia. Int. J. Syst. Evol. Microbiol..

[47] Vincent J.M. (1970). A manual for the practical study of the root-nodule bacteria. IBP Handbook No. 15.

[48] Vinuesa P., Rojas-Jiménez K., Contreras-Moreira B., Mahna S.K., Prasad B.N., Moe H., Selvaraju S.B., Thierfelder H., Werner D. (2008). Multilocus sequence analysis for assessment of the biogeography and evolutionary genetics of four *Bradyrhizobium* species that nodulate soybeans on the asiatic continent. Appl. Environ. Microbiol..

[49] Wade T.K., Le Quéré A., Laguerre G., N’Zoué A., Ndione J.-A., doRegoJ F., Sadio O., Ndoye I., Neyra M. (2014). Eco-geographical diversity of cowpea bradyrhizobia in Senegal is marked by dominance of two genetic types. Syst. Appl. Microbiol..

[50] Xu L.M., Ge C., Cui Z., Li J., Fan H. (1995). *Bradyrhizobium liaoningense* sp. nov., isolated from the root nodules of soybeans. Int. J. Syst. Evol. Microbiol..

[51] Yao Z.Y., Kan F.L., Wang E.T., Wei G.H., Chen W.X. (2002). Characterization of rhizobia that nodulate legume species of the genus Lespedeza and description of *Bradyrhizobium yuanmingense* sp. nov. Int. J. Syst. Evol. Microbiol..

[52] Zeigler D.R. (2003). Gene sequences useful for predicting relatedness of whole genomes in bacteria. Int. J. Syst. Evol. Microbiol..

[53] Zhang Y.M., Li Y., Chen W.F., Wang E.T., Tian C.F., Li Q.Q., Zhang Y.Z., Sui X.H., Chen W.X. (2011). Biodiversity and biogeography of rhizobia associated with soybean plants grown in the North China Plain. Appl. Environ. Microbiol..

