# Supplementary material for: Identification and distribution of microsymbionts associated with soybean nodulation in Mozambican soils
Source: Syst Appl Microbiol. 2018 Sep;41(5):506–15. doi: 10.1016/j.syapm.2018.05.003 (PMC6150944; doi:10.1016/j.syapm.2018.05.003)
Supplement: Supplementary file 1 [file mmc1.docx]

Identification and distribution of microsymbionts associated with soybean nodulation in Mozambican soils

Cynthia Gyogluu^1^, Sanjay K Jaiswal ^2^, Stephen K Boahen^3^, and Felix. D Dakora^2^

^1^Department of Crop Sciences, and ^2^Department of Chemistry, Tshwane University of Technology, Pretoria, South Africa; ^3^International Institute of Tropical Agriculture, Nampula, Mozambique

**Table S1** Primers and PCR temperature profiles used for DNA amplification.

| **Gene name** | **Primer Sequence (5’ – 3’)** | **Temperature profile** | **References** | |
| --- | --- | --- | --- | --- |
|  |  |  | |  |
| 16S rDNA | fD1 (9-29)AGAGTTTGATCCTGGCTCAG  rD1 (1474-1494)CTTAAGGAGGTGATCCAGCC | 5 min 95 ̊C, 35 X (1 min 95 ̊C, 1 min 55 ̊C, 1 min 72 ̊C), 10 min 72 | | [1] |
| *gln*ll | 13f-AAGCTCGAGTACATCTGGCTCGACGG  681r-SGAGCCGTTCCAGTCGGTGGTGTCG | 2 min 95 ̊C, 35 X (45 s 95 ̊C, 30 s 65 ̊C, 90 s 72 ̊C), 10 min 72 ̊ C | | [2] |
| *gyr*B | 343f-TTCGACCAGAAYTCCTAYAAGG  1043r-AGCTTGTCCTTSGTCTGCG | 10 min 95 ̊C, 35 X (30 s 94 ̊C, 30 s 58 ̊C, 1 min 72 ̊C), 10 min 72 ̊ C | | [3] |
| *nifH* | 5’TACGGNAARGGSGGNATCGGCAA3’  5’AGCATGTCYTCSAGYYTCNTCCA3’ | 5 min 94 ̊C, 20 X [30 s 94 ̊C, 30 s 65 ̊C (-0.5 ̊C\cycle),90 s 72 ̊C], 25 X (30 s 94 ̊C, 30 s 55 ̊C, 90 s 72 ̊C), 10 min 72 ̊ C | | [4] |
| *nodC* | 5’GTC GAT TGC MRG TCA AGA CTA CG 3’  5’GCC AGG TCT IGT TGC GAT TGC TC 3’ | 30 s 94 ̊C, 40 X [30s 94 ̊C, 1 min 57 ̊C, 30s 72 ̊C], 5 min 72 ̊C | | [5] |

[1] Weisburg, W.G., Barns, S.M., Pelletier, D.A., Lane, D.J. (1991) 16S ribossomal DNA amplification for phylogenetic study. J.

Bacteriol. 173, 697-703

[2] Stępkowski, T., Żak, M., Moulin, L., Króliczak, J., Golińska, B., Narożna, D., ... Mądrzak, C.J. (2011) *Bradyrhizobium canariense*

and *Bradyrhizobium japonicum* are the two dominant *Rhizobium* species in root nodules of lupin and serradella plants growing in

Europe. Syst. Appl. Microbiol. 34, 368-375.

[3] Marek-Kozaczuk, M., Leszcz, A., Wielbo, J., Wdowiak-Wróbel, S., Skorupska, A. (2013) *Rhizobiumpisi*sv. *trifolii* K3. 22

harboring nod genes of the *Rhizobium leguminosarum* sv. *trifolii* cluster. Syst. Appl.Microbiol. 36(4), 252-258

[4] Nzoué, A., Miché, L., Klonowska, A., Laguerre, G., de Lajudie, P., Moulin, L. (2009) Multilocus sequence analysis of bradyrhizobia isolated from Aeschynomene species in Senegal. Syst. Appl. Microbiol. 32(6), 400-412.

[5] Sterner, J.P., Parker, M.A. (1999) Diversity and relationships of bradyrhizobia from *Amphicarpaea bracteata* based on partial *nod*

and ribosomal sequences. Syst. Appl. Microbiol. 22(3), 387-392.

**Table S2** Accession numbers of test soybean isolate sequences used in the study

|  |  | Loci | | | | | | |  |
| --- | --- | --- | --- | --- | --- | --- | --- | --- | --- |
| Soybean isolate |  | 16s |  | *gln*II |  | *gyr*B |  | *nif*H | *nodC* |
| TUTNFM2A1 |  | KT380043 |  | KP410690 |  | KR091912 |  | KR491961 | MH041436 |
| TUTMCJ4B |  | KT380044 |  | KP410691 |  | KR491978 |  | KR491962 | MH041431 |
| TUTDAIAP3B |  | MH041425 |  | MF150158 |  | KR4919981 |  | KR491965 | MH041437 |
| TUTDAIAP8B |  | KR491954 |  | KP410693 |  | KR4919982 |  | KR491967 | - |
| TUTMIITA5A1 |  | MH041427 |  | KP410694 |  | KR4919983 |  | KR491969 | MH041428 |
| TUTMFJ1A1 |  | KR491956 |  | KP410695 |  | KR4919984 |  | KR491970 | - |
| TUTRAH5B2 |  | KR491957 |  | KP410696 |  | KR491986 |  | KR491975 | MH041433 |
| TUTRLR3B |  | MF140378 |  | KP410697 |  | KR491987 |  | KR491976 | - |
| TUTMIITA5A2 |  | KR491951 |  | KP410699 |  | KR491979 |  | KR491963 | MH041443 |
| TUTMFJ2B |  | KR491952 |  | KP410700 |  | KR491980 |  | KR491964 | MH041430 |
| TUTMCJ7B |  | KT380045 |  | KP410701 |  | KR491985 |  | KR491973 | MH041438 |
| TUTRAB5B1 |  | KR491959 |  | MF150159 |  | MF150186 |  | KR491977 | MH041440 |
| TUTMCJ5B |  | MH041426 |  | MF150160 |  | MF150185 |  | KR491972 | MH041439 |
| TUTNFM1A |  | MF140377 |  | MF150153 |  | MF150181 |  | MF150187 | - |
| TUTRAH8A |  | MF140379 |  | MF150155 |  | MF150183 |  | MF150188 | - |
| TUTMJM5 |  | MF140380 |  | MF150156 |  | MF150184 |  | MF150189 | MH041444 |

**Table S3** Total RFLP pattern and restriction pattern type of 16S-rDNA

|  |  | 16S rDNA restriction pattern | | |  |  |
| --- | --- | --- | --- | --- | --- | --- |
|  | **Isolate** | ***Hae*III** | ***Msp*I** | ***Hpa*II** | **Soil location** | **Soybean variety** |
| Cluster I | TUTNFM2A1 | HA1 | M1 | Hp1 | Ntengo | TGx1740-2F |
|  | TUTMFJ4 | HA1 | M1 | Hp1 | Muteq | TGx1951-3F |
|  | TUTRAH8A | HA1 | M5 | Hp1 | Ruace | Serenade |
|  | TUTRAB5B1 | HA3 | M5 | Hp1 | Ruace | Serenade |
|  | TUTMIITA5A2 | HA3 | M1 | Hp3 | Muteq | TGx1937-1F |
|  | TUTMFJ3B | HA3 | M1 | Hp4 | Muteq | TGx1951-3F |
|  | TUTMFJ2B | HA3 | M1 | Hp4 | Muteq | TGx1951-3F |
|  | TUTMJM6A | HA3 | M1 | Hp4 | Magige | Santa Rosa |
|  | TUTMIITA4A | HA3 | M1 | Hp4 | Muteq | TGx1937-1F |
|  | TUTMFJ1A1 | HA3 | M1 | Hp5 | Muteq | TGx1951-3F |
|  | TUTM19373A | HA3 | M1 | Hp5 | Muriase | TGx1937-1F |
|  | TUTM19043A | HA3 | M1 | Hp5 | Muriase | TGx1904-6F |
|  | TUTRAH5B2 | HA3 | M4 | Hp5 | Ruace | Serenade |
|  | TUTRSRH8B | HA3 | M7 | Hp5 | Ruace | Serenade |
|  | TUTRJN5A | HA3 | M9 | Hp5 | Ruace | Serenade |
|  | TUTNFM1A | HA3 | M9 | Hp8 | Ntengo | TGx1740-2F |
|  | TUTLBC1B | HA3 | M9 | Hp8 | Livirange | TGx1908-8F |
|  | TUTMCJ9A | HA3 | M15 | Hp16 | Muteq | TGx1740-2F |
|  | TUTMCJ5B | HA3 | M13 | Hp16 | Muteq | TGx1740-2F |
|  |  |  |  |  |  |  |
| Cluster II | TUTMJM5 | HA6 | M11 | Hp11 | Magige | TGx1937-1F |
|  | TUTDAIAP3B | HA6 | M12 | Hp11 | Angonia | TGx1740-2F |
|  | TUTMCJ4B1 | HA6 | M13 | Hp11 | Muteq | TGx1951-3F |
|  | TUTDAIAP8B | HA7 | M13 | Hp13 | Angonia | TGx1740-2F |
|  | TUTDAIAP1A | HA8 | M14 | Hp14 | Angonia | TGx1740-2F |
|  | TUTDAIAP2A1 | HA7 | M14 | Hp12 | Magige | Santa Rosa |
|  | TUTMIITA5A | HA7 | M8 | Hp12 | Muteq | TGx1937-1F |
|  | TUTRJN3A1 | HA9 | M8 | Hp12 | Ruace | Serenade |
|  | TUTNSN2A | HA9 | M8 | Hp12 | Ntengo | TGx1904-6F |
|  | TUTLBC2B | HA9 | M17 | Hp12 | Livirange | TGx1908-8F |
|  | TUTMCJ10B | HA4 | M16 | Hp17 | Muteq | TGx1740-2F |
|  | TUTRLR4B2 | HA4 | M8 | Hp7 | Ruace | TGx1908-8F |
|  | TUTSFD1A | HA4 | M8 | Hp7 | Serra | Safari |
|  | TUTNFM3B | HA9 | M13 | Hp9 | Ntengo | TGx1740-2F |
|  | TUTNSN3B | HA9 | M8 | Hp12 | Ntengo | TGx1904-6F |
|  |  |  |  |  |  |  |
| Cluster III | TUTMCJ4B | HA2 | M2 | Hp2 | Muteq | TGx1740-2F |
|  | TUTMIITA5A1 | HA2 | M10 | Hp15 | Muteq | TGx1937-1F |
|  | TUTMCJ5B1 | HA2 | M3 | Hp6 | Muteq | TGx1740-2F |
|  | TUTRAB2B | HA2 | M6 | Hp6 | Ruace | Serenade |
|  | TUTRSRH9A | HA2 | M6 | Hp6 | Ruace | Serenade |
|  | TUTRLR3B | HA2 | M6 | Hp6 | Ruace | TGx1908-8F |
|  | TUTNSN3B1 | HA2 | M6 | Hp6 | Ntengo | TGx1904-6F |
|  | TUTMFJ3BG | HA5 | M10 | Hp10 | Muteq | TGx1951-3F |
|  |  |  |  |  |  |  |
|  | TUTMCJ7B | HA10 | M18 | Hp18 | Muteq | TGx1740-2F |

**Table S4** Information of nucleotide sequences participated in phylogenetic analysis

| **Locus** | **No. of strains used for tree construction** | **Nucleotide sequence information** | | | | **Total*** | **Frequency**  **T/C/A/G**  **(%)** |
| --- | --- | --- | --- | --- | --- | --- | --- |
|  |  | **Cnoserved (C)** | **Variables (V)** | **Parsimony-informative (Pi)** | **Singleton (S)** |  |  |
| *16S rDNA* | 61 | 596 (80.50) | 140 (18.91) | 22 (2.97) | 117 (15.81) | 740 | 20.1/21.8/26.1/32.0 |
| *gln*II | 52 | 237 (62.70) | 141 (37.30) | 100 (26.46) | 41 (10.85) | 378 | 20.5/30.1/16.9/32.6 |
| *gyr*B | 47 | 294 (54.44) | 240 (63.49) | 142 (26.30) | 98 (18.15) | 540 | 20.6/31.1/16.0/32.3 |
| Concatenated  (*glnII*+ *gyr*B) | 33 | 544 (59.26) | 368 (40.08) | 227 (24.73) | 141 (15.36) | 918 | 20.6/30.6/16.3/32.4 |
| *nif*H | 47 | 165 (60.22) | 109 (39.78) | 86 (31.39) | 23 (8.39) | 274 | 19.7/34.0/20.0/26.4 |
| *nodC* | 32 | 85 (42.5) | 111 (55.5) | 92 (46) | 19 (9.5) | 200 | 18.0/26.8/23.1/32.1 |

*number of sites used for tree construction

Fig. S1: Phylogenetic tree based on glnII sequences generated by Neighbour-joining algorithm. Bootstrap values (1000 replicates) are indicated above the branches.

*Bradyrhizobium* *japonicum*^T^ (AF169582)

*Bradyrhizobium* *diazoefficiens* USDA 110^T^ ( CP011360.1)

*Bradyrhizobium* *betae* LMG 21987^T^ (AB353733.1)

*Bradyrhizobium* *ganzhouense* RITF807^T^ (JX277111)

*Bradyrhizobium* *cytisi* CTAW1^T^ (GU001594)

*Bradyrhizobium* *rifense* CTAW7^T^ (GU001604)

*Bradyrhizobium* *huanghuaihaiense* CCBAU 23303^T^ (HQ231639)

*Bradyrhizobium* *iriomotense*^T^ (AB300995.1)

*Bradyrhizobium* *ingae* BR 10250^T^ (KF927067)

*Bradyrhizobium* *liaoningense* bv. *glycinearum* LMG 18230^T^ (AY386775)

*Bradyrhizobium* *ottawaense* OO99^T^ (HQ587750)

*Bradyrhizobium* *arachidis* CCBAU 051107^T^ (HM107251)

*Bradyrhizobium* *subterraneum* 60 2-1^T^ (KM378485.1)

*Bradyrhizobium* *yuanmingense* CCBAU 10071^T^ (AY386780)

*Bradyrhizobium* *daqingense* CCBAU 15774^T^ (HQ231301)

**TUTDAIAP3B**

*Bradyrhizobium* *vignae* 7-2^T^ (KM378443)

*Bradyrhizobium* *manausense* BR 3351^T^ (KF785986)

*Bradyrhizobium* *guangdongense* CCBAU 51649^T^ (KC509023)

*Bradyrhizobium* *kavangense* 14-3^T^ (KM378446)

*Bradyrhizobium* *guangxiense* CCBAU 53363^T^ (KC509033)

*Rhizobium* *lusitanum* P1-7^T^ (EF639841.1)

*Bradyrhizobium* *oligotrophicum* LMG 10732^T^ (JQ619233)

*Bradyrhizobium* *denitrificans* LMG 8443^T^ (HM047121)

*Bradyrhizobium* *erythrophlei* CCBAU 53325^T^ (KF114693)

*Bradyrhizobium* *viridifuturi* SEMIA 690^T^ (KR149131)

*Bradyrhizobium* *pachyrhizi* PAC48^T^ (FJ428201)

*Bradyrhizobium* *ferriligni* CCBAU 51502^T^ (KJ818099)

*Bradyrhizobium* embrapense SEMIA 6208^T^ (GQ160500)

*Bradyrhizobium* *tropiciagri* SEMIA 6148 ^T^ (FJ391048)

*Bradyrhizobium* *elkanii* USDA 76 ^T^ (AY599117)

**TUTRAH8A**

**TUTMJM5**

**TUTNFM1A**

**TUTRAB5B1**

**TUTRLR3B**

**TUTRAH5B2**

**TUTNFM2A1**

**TUTMCJ7B**

**TUTMCJ4B**

**TUTMIITA5A2**

**TUTMFJ2B**

**TUTDAIAP8B**

**TUTMIITA5A1**

**TUTMFJ1A1**

**TUTMCJ5B**

*Bradyrhizobium* *valentinum* LmjM3^T^ (JX518575)

*Bradyrhizobium* *retamae* Ro19^T^ (KC247108)

*Bradyrhizobium* *icense* LMTR 13^T^ (KF896175)

*Bradyrhizobium* *lablabi* CCBAU 23086^T^ (GU433498)

*Bradyrhizobium* *jicamae* PAC68^T^ (FJ428204)

*Bradyrhizobium* *paxllaeri* LMTR 21^T^ (KF896169)

100

95

93

63

85

51

53

68

79

73

55

98

71

99

83

66

90

0.01

II

I

Fig. S3: Phylogenetic tree based on *gyrB* sequences generated by Neighbour-joining algorithm. Bootstrap values (1000 replicates) are indicated above the branches.

**TUTMCJ5B**

**TUTRAB5B1**

**TUTMJM5**

**TUTRAH8A**

**TUTNFM1A**

**TUTRLR3B**

**TUTRAH5B2**

**TUTMCJ7B**

**TUTMFJ1A1**

*Bradyrhizobium* *elkanii* LMG 6134^T^ (AM418800.1)

**TUTNFM2A1**

**TUTMCJ4B**

**TUTMIITA5A2**

**TUTMFJ2B**

**TUTMIITA5A1**

**TUTDAIAP3B**

**TUTDAIAP8B**

*Bradyrhizobium* *pachyrhizi* PAC 48^T^ (HQ873310)

*Bradyrhizobium* *enbrapense* SEMIA 6208^T^ (HQ634891)

*Bradyrhizobium* *ferriligni* CCBAU 51502^T^ (KJ818102)

*Bradyrhizobium* *viridifuturi* SEMIA 690^T^ (KR149134)

*Bradyrhizobium* *tropiciagri* SEMIA 6148^T^ (HQ634890)

*Bradyrhizobium* *erythrophlei* CCBAU 53325^T^ (KF114717)

*Bradyrhizobium* *retamae* Ro19^T^ (KF962698)

*Bradyrhizobium* *icense* LMTR 13^T^ (KF896201)

*Bradyrhizobium* *jicamae* PAC 68^T^ (HQ873309)

*Bradyrhizobium* *paxllaeri* LMTR 21^T^ (KF896195)

*Bradyrhizobium* *lablabi* CCBAU 23086^T^ (KF962696)

*Bradyrhizobium* *ottawaense* OO99^T^ (HQ873179)

*Bradyrhizobium* *diazoefficiens* USDA 110^T^ (CP011360.1)

*Bradyrhizobium* *betae* LMG 21987^T^ (FM253217.1)

*Bradyrhizobium* *canariense* LMG 22265^T^ (FM253220.1)

*Bradyrhizobium* *ganzhouense*^T^ (KP420022)

*Bradyrhizobium* *rifense* CTAW71^T^ (KC569466)

*Bradyrhizobium* *huanghuaihaiense* CCBAU 23303^T^ (KF962695)

*Bradyrhizobium* *manausense* BR 3351^T^ (KF786000.1)

*Bradyrhizobium* *japonicum* LMG 6138^T^ (AM418801.1)

*Bradyrhizobium* *guangdongense* CCBAU 51649^T^ (KC509072)

*Bradyrhizobium* *ingae* BR 10250^T^ (KF927079)

*Bradyrhizobium* *liaoningense* LMG 18230^T^ (FM253223.1)

*Bradyrhizobium* *kavangense* 14-3^T^ (KX661397.1)

*Bradyrhizobium* *yuanmingense* LMG 21827^T^ (FM253226.1)

*Bradyrhizobium* *daqingense* CCBAU 15774^T^ (KF962694)

*Bradyrhizobium* *guangxiense* CCBAU 53363^T^ (KC509082)

*Bradyrhizobium* *subterraneum* 58 2-1^T^ (KX661396.1)

*Bradyrhizobium* *denitrificans* LMG 8443^T^ (FM253239.1)

*Rhizobium* *lusitanum* P1-7^T^ (KC293525.1)

96

100

100

100

65

51

79

64

74

98

93

100

0.05

I

II
